# A systematic review of adherence to oral pre-exposure prophylaxis for HIV – how can we improve uptake and adherence?

**DOI:** 10.1186/s12879-018-3463-4

**Published:** 2018-11-16

**Authors:** David Sidebottom, Anna Mia Ekström, Susanne Strömdahl

**Affiliations:** 1 Department of Public Health Sciences, Karolinska Institutet, Stockholm, Sweden; 2 Department of Medical Sciences, Section of Infectious Diseases, Uppsala University, Uppsala, Sweden; 3 Department of Infectious Diseases, Karolinska University Hospital, Stockholm, Sweden

**Keywords:** HIV, Pre-exposure prophylaxis, HIV prevention, Systematic review, Medication adherence, Antiviral drug resistance

## Abstract

**Introduction:**

Oral pre-exposure prophylaxis (PrEP) is an effective strategy to reduce the risk of HIV transmission in high risk individuals. However, the effectiveness of oral pre-exposure prophylaxis is highly dependent on user adherence, which some previous trials have struggled to optimise particularly in low and middle income settings. This systematic review aims to ascertain the reasons for non-adherence to pre-exposure prophylaxis to guide future implementation.

**Methods:**

We performed structured literature searches of online databases and conference archives between August 8, 2016 and September 16, 2017. In total, 18 prospective randomized control trials and implementation studies investigating oral pre-exposure prophylaxis were reviewed. A structured form was used for data extraction and findings summarized regarding efficacy, effectiveness, adherence and possible reasons for non-adherence.

**Results:**

Adherence varied between differing populations both geographically and socioeconomically. Common reasons for non-adherence reported over multiple studies were; social factors such as stigma, low risk perception, low decision making power, an unacceptable dosing regimen, side effects, and the logistics of daily life. Oral pre-exposure prophylaxis with included antiviral regimens was not associated with a high risk of antiviral resistance development in the reviewed studies.

**Conclusion:**

Our findings indicate that oral pre-exposure prophylaxis should be delivered within a holistic intervention, acknowledging the other needs of the targeted demographic in order to maximise acceptability. Socioeconomic factors and poor governmental policy remain major barriers to widespread implementation of pre-exposure prophylaxis.

## Background

In the face of barriers due to policy, stigma, and culture, progress is being made in the struggle against HIV. Antiretroviral therapy (ART) is undergoing rapid global scale-up with the 90–90-90 2020 United Nations (UN) target in sight, reaching 46% global coverage in 2015 compared to less than 10% the decade before [1]. This translates into a 26% reduction in global AIDS-related deaths since 2010. Additionally, with the efficacy of treatment as prevention demonstrated in 2011 [2], ART holds the potential to reduce HIV incidence beyond the 36.7 million people already infected. However, despite these advances, the Joint United Nations Programme on HIV/AIDS (UNAIDS) notes that recent headway in HIV incidence reduction has slowed “alarmingly”, and that disparities in progress are widening for certain key populations such as young women, sex workers, people who inject drugs (PWID) and men who have sex with men (MSM) [1].

It is within these key populations that the burden of HIV is disproportionately carried. The risk of HIV acquisition versus the general population is 10 times greater in sex workers and 24 times greater in PWID and MSM [3], although analysis reveals large diversity between regions. In Western Europe and North America, 49% of new infections occur within the MSM population and 15% in PWID, whereas in Eastern Europe and Central Asia the figures are 6 and 51% respectively [1]. This variation reflects the diverse burden of stigma and discrimination borne by these populations [4]. Same-sex acts are illegal in 72 (37%) UN states, and punishable by the death penalty in 13 (6%) [4], just one example of the many additional challenges faced by individuals and organisations battling HIV. Effective prevention strategies to combat HIV are desperately needed by these hidden populations, none more so than transgender women (TGW), who have nearly 49 times greater odds of HIV acquisition than the general population [5].

In 2010 iPrEx became the first randomised controlled trial (RCT) to demonstrate the efficacy of pre-exposure prophylaxis (PrEP) in MSM, finding a 44% risk reduction in the experimental group receiving daily oral tenofovir-emtricitabine (TDF-FTC) as compared to placebo [6]. This success has since been replicated in several further studies encompassing both daily [7–9] and on-demand regimens [10], fuelling global excitement over this novel strategy. Following this data, PrEP is recommended for implementation among MSM by the World Health Organisation (WHO) and the Centres for Disease Prevention and Control (CDC) [11, 12]. However, failures have been observed in some at-risk groups, most notably heterosexual women [13, 14].

Previous literature notes that adherence is a critical link in the wider PrEP continuum, and that the success of PrEP intervention rides on its ability to maintain good adherence within the cohort under investigation [15, 16]. In 2013, a nested sub-study of the Partners trial found that high (> 80%) PrEP adherence was associated with 100% PrEP efficacy (95% CI 83.7 to 100%) [17]. Conversely, in 2015 the VOICE trial failed to demonstrate PrEP clinical effectiveness in young African women [14], where only 30% of quarterly plasma samples contained a detectable level of TDF. Whilst a number of reviews exist concerning various aspects of PrEP we conducted this global systematic review to assess adherence to oral PrEP in the context of the reported efficacy. We also aimed to discuss the reasons for non-adherence in detail to guide comprehensive PrEP implementation programming in the future.

## Methods

### Search strategy and inclusion criteria

**Fig. 1:**
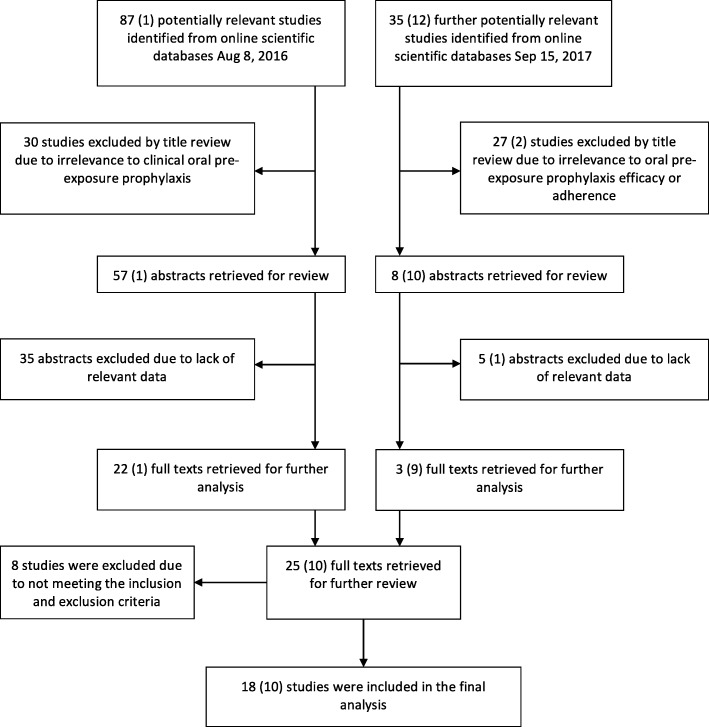
Flow diagram illustrating review process [64]. Numbers in brackets represent conference abstracts

The Population, Intervention, Comparison, Outcome (PICO) framework was used to develop the search strategy. The population was defined as all individuals ‘at risk’ of HIV acquisition that have been studied regarding PrEP. Eligible studies comprised of prospective RCTs and implementation studies that examined efficacy, effectiveness or adherence. Both studies reporting oral TDF and TDF-FTC as the intervention were included as this distinction has not been shown to be clinically important [7, 18]. Daily, event and time driven regimens were all eligible (Fig. 1). The outcomes assessed were efficacy and adherence. For adherence all measurements were included. All comparison and no-comparison trials were included. In practice the ‘at risk’ population is reflected in trial recruitment criteria, so was not specified in our search. No restrictions were imposed regarding geographical location, sex or gender, sexual preference, or dosing regimen. Only English language trials discussing oral PrEP efficacy, effectiveness or adherence in detail were included.

We performed online searches in Ovid Medline (without revisions, 1996 to current), Web of Science, EMBASE and the Cochrane Library. An initial search was conducted in August 2016, and repeated in September 2017. In addition, we searched conference abstracts from the AIDS Conference, International AIDS Society Conference and the Conference on Retroviruses and Opportunistic Infections via their online archives.

Our search utilised a combination of medical subject heading terms (denoted by appended ‘/’) and keywords as follows; (Pre-Exposure Prophylaxis/ OR PrEP OR chemoprophyla* OR antiretroviral prophyla*) AND (HIV/ OR HIV-1/ OR Anti-HIV Agents/). Results were limited to ‘human’ and ‘clinical trial’, from 2010 to ‘current’ as the first PrEP RCT was published in 2010.

### Screening and data extraction

Published studies were identified through the search strategy described above, and titles were screened for relevance. Abstracts were further screened for eligibility and downloaded for further analysis when inclusion criteria were met. Identified articles were critically appraised using a checklist [19] to assess methodology prior to inclusion into the systematic review. Attention was paid to randomisation and blinding adequacy, allocation concealment and loss to follow up.

An initial online search on August 8, 2016 located 87 potentially relevant papers, and one conference abstract. A repeated search on September 15, 2017 located an additional 35 papers, and 12 conference abstracts. In total, 18 papers and 10 conference abstracts were found. DS extracted the data using a structured form regarding study design and population, geographical location and time, sample size, follow-up time, drug regimen, efficacy measurement, and adherence measurements. An additional 5 papers reported on qualitative exploration of factors affecting PrEP adherence. Authors were contacted by email if clarification was required.

All adherence measurements used were included, as defined in cited literature. Detection of TDF and/or FTC in plasma is highly concordant with the presence of TDF/FTC active metabolites within HIV-1 target cells, which provides protection from HIV [6, 20]. Tenofovir diphosphate levels, measured through dried blood spot testing, is increasingly used as an intrusive biomarker of long-term PrEP adherence, due to its long half-life of 17 days [15, 21]. However, a variety of soft adherence measures are also used (pill count, self-report, medication event monitoring systems (MEMS). Where adherence at multiple time points was available, the latest measurement was taken, as maintaining prolonged adherence to PrEP throughout the duration of possible exposure to HIV is arguably of most clinical interest. Efficacy data was also extracted from the literature, as this important outcome is best understood in the context of reported adherence.

## Results

Study design is illustrated in Fig. 1. Randomised controlled trials have evaluated oral PrEP in a variety of geographical and sociological settings. The characteristics of included trials are displayed in Table 1.

**Table 1:** Overview of included studies examining the efficacy, effectiveness, and adherence of oral PrEP

Characteristics									Number of incident HIV infections		
Year	Study name	Geographical location	Population	Sample size	Total follow-up time (person-years)	Design	Regimen	Drug	Study drug	Placebo/ comparator	Total
2010	iPrEx [6]	Global	MSM/TGW	2499	3324	RDBPCT	Daily	TDF-FTC	36	64	100
2012	Partners study [7]	Kenya and Uganda	Heterosexual HIV-discordant couples	4758	7820	RDBPCT	Daily	TDF-FTC	13	52	82
								TDF	17		
2012	TDF2 [8]	Botswana	Heterosexual	1219	1563	RDBPCT	Daily	TDF-FTC	9	24	33
2012	FEM-PrEP [13]	Kenya, Tanzania and South Africa	Heterosexual females	2120	–	RDBPCT	Daily	TDF-FTC	33	35	68
2012	Kenya safety and adherence study [38]	Kenya	MSM and Female sex workers	72	–	RDBPCT	Daily	TDF-FTC	0	1	1
							Time-driven		0		
2013	Partners adherence substudy [17]	Kenya and Uganda	Heterosexual HIV-discordant couples	1147	807	Convenience sub-cohort of a RDBPCT	Daily	TDF-FTC	0	14	14
								TDF	0		
2013	Bangkok tenofovir study [31]	Bangkok	PWID	2413	9665	RDBPCT	Daily	TDF	17	33	50
2013	Uganda safety and adherence study [35]	Uganda	Heterosexual HIV-discordant couples	72	–	RDBPCT	Daily	TDF-FTC	0	0	0
							Time-driven		0	0	
2013	ATN 082 (Project PrEPARE) [54]	United States	Young MSM	58	–	RBPCT	Daily	TDF-FTC	0	0	0
								No pill	0		
2014	iPrEx extension [15]	Global	MSM/TGW	1603 (1225 received)	–	Open Label	Daily	TDF-FTC	28	13	41
2015	VOICE [14]	South Africa, Uganda, Zimbabwe	Heterosexual females	3019	4253	RCT	Daily	TDF-FTC	61	60	173
								TDF	52		
2015	HPTN 067/ADAPT^a^ [36]	South Africa	Heterosexual females	191	–	RCT with different regimens as comparators	Daily	TDF-FTC	1	N/A	5
							Time-driven		2		
							Event-driven		2		
2015	Generating adherence Philadelphia [50]	United States	Young MSM of colour	23	7.5	Observational	Daily	TDF-FTC	0	N/A	0
2015	PROUD [9]	United Kingdom	MSM	544	465	RCT with a 1 year deferred group as comparator	Daily	TDF-FTC	3	20	23
2015	IPERGAY [10]	France and Canada	MSM/TGW	400	431	RDBPCT	Event-driven	TDF-FTC	2	14	16
2016	Bangkok MSM^a^ [55]	Thailand	MSM /TGW	168	–	Observational	Daily	TDF-FTC	0	N/A	0
2016	Permanente Cohort [24]	USA	At-risk	972	850	Open label	Daily	TDF-FTC	0	2 Off-PrEP	2
2016	The Demo Project [23]	USA	MSM/TGW	557	481	Open label	Daily	TDF-FTC	1	1 Off-PrEP	2
2017	SPARK^a^ [57]	United States	MSM	301	–	Open Label	Daily	TDF-FTC	–	–	–
2017	IPERGAY extension [22]	France/Canada	MSM/TGW	361	518	Open label	Event-driven	TDF-FTC	0	1 Off-PrEP	1
2017	Short term PrEP Mozambique^a^ [25]	Mozambique	Heterosexual females	74	7.4	Open label	Daily	TDF-FTC	0	N/A	1
2017	Parisian MSM^a^ [26]	France	MSM	785	215	Open label	Daily & Event-driven	TDF-FTC	3	N/A	3
2017	PRELUDE^a^ [27, 28]	Australia	Gay/bisexual males	317	381	Open label	Daily	TDF-FTC	0	N/A	0
2017	PROUD adherence^a^ [29]	UK	MSM	544 enrolled (481 initiated)	1253	Open label	Daily	TDF-FTC	10	N/A	10
2017	Pluspills^a^ [30]	South Africa	Adolescents	148	131	Open label	Daily	TDF-FTC	0	1 Off-PrEP	1
2017	Brazil Demo^a^ [65]	Brasil	MSM/TGW	450	389	Open label	Daily	TDF-FTC	0	2 Off-PrEP	2

^a^ Abstract available only. *MSM* men who have sex with men, *TGW* transgender women, *PWID* people who inject drugs, *RCT* randomised controlled trial, *RDBPCT* randomised, double blinded, placebo controlled trial, *TDF* tenofovir, *FTC* emtricitabine

### Adherence

Table 2 describes reported study adherence by various measures. Wide disparity exists between soft (self-report, pill count, medication event monitoring system) and intrusive (levels of TDF and/or FTC in plasma samples, or tenofovir diphosphate measured in dried blood spots) measures of adherence. In all measured cases, a higher proportion of non-seroconverters have detectable plasma TDF than seroconverters. Two trials which failed in young African women are associated with poor adherence. Only 24% of non-seroconverters had detectable TDF in FEM-PrEP [13], and 29% in VOICE [14]. In contrast, results from a series of recent open label papers and abstracts suggest high adherence in a variety of real-world settings [22–30].

**Table 2:** Adherence to oral PrEP by different measures used

Characteristics					Adherence				
Year	Study name	Geographical location	Population	Regimen	Any detectable plasma drug (TDF or FTC) (%)		Self-report (%)	Pill count (%)	MEMS (%)
					HIV – (non-seroconverters)	HIV + (seroconverters)			
2010	iPrEx [6]	Global	MSM/TGW	Daily	51	9	95	> 90	–
2012	Partners study [7]	Kenya and Uganda	Heterosexual HIV-discordant couples	Daily	82	31	–	92	–
2012	TDF2 [8]	Botswana	Heterosexual	Daily	80	50	94	84	–
2012	FEM-PrEP [13]	Kenya, Tanzania, South Africa	Heterosexual females	Daily	24	15	95	88	–
2012	Kenya safety and adherence study [38]	Kenya	MSM and female sex workers	Daily	–	–	–	–	83% (IQR 63 to 92)
				Time-driven			100		55 (pre-coital), 26 (post-coital)
2013	Partners adherence substudy [17]	Kenya and Uganda	Heterosexual HIV-discordant couples	Daily	–	–	–	99	97
2013	Bangkok tenofovir study [31]	Bangkok	PWID	Daily	67	39	94	–	–
2013	Uganda safety and adherence study [35]	Uganda	Heterosexual HIV-discordant couples	Daily	–		–	–	97
				Time-driven			100		91 (pre-coital) 45 (post-coital)
2013	ATN 082 (Project PrEPARE) [54]	United States	Young MSM	Daily	20		62	–	–
2014	iPrEx extension [15]	Global	MSM/TGW	Daily	71		85 ^c^	–	–
2015	VOICE [14]	South Africa, Uganda, Zimbabwe	Heterosexual females	Daily	29supp		87 (via computer), 90 (face to face)	88	–
					30		87 (via computer), 91 (face to face)	84	
2015	Generating adherence Philadelphia [50]	United States	Young MSM of colour	Daily	–		–	72	–
2015	HPTN 067/ADAPT^a^ [36]	South Africa	Heterosexual females	Daily	68		–	–	76
				Time-driven	56				65
				Event-driven	53				53
2015	PROUD [9]	United Kingdom	MSM	Daily	100 ^c^		–	–	–
2015	IPERGAY [10]	France and Canada	MSM/TGW	Event-driven	87	0	29 (suboptimal), 43 (optimal) ^b^	–	–
2016	Bangkok MSM^a^ [55]	Thailand	MSM/TGW	Daily	–		9.8 (complete adherence)	–	–
2016	Permanente Cohort [24]	USA	At-risk	Daily	–		–	92	–
2016	The Demo Project [23]	USA	MSM/TGW	Daily	80 ^d^		–	82	–
2017	SPARK^a^ [57]	United States	MSM	Daily	90		–	–	–
2017	IPERGAY extension [22]	France/Canada	MSM/TGW	Event-driven	71 ^e^	0	24 (suboptimal), 50 (optimal)	–	–
2017	Short term PrEP Mozambique^a^ [25]	Mozambique	Heterosexual females	Daily	76				–
2017	Parisian MSM^a^ [26]	France	MSM	Daily & Event-driven	83		–	–	–
2017	PRELUDE^a^ [27, 28]	Australia	Gay/bisexual males	Daily	51 ^d^		–	–	–
2017	PROUD adherence^a^ [29]	UK	MSM	Daily	–		98	–	–
2017	Pluspills^a^ [30]	South Africa	Adolescents	Daily	38		–	92	–
2017	Brazil Demo^a^ [65]	Brasil	MSM/TGW	Daily	74		–	–	–

^a^ Abstract available only, ^b^ At most recent sexual encounter, ^c^ Of participants reporting good adherence, ^d^ Dried blood spot concentration, ^e^Only 33% of participants had plasma TDF concentrations consistent with taking > 4 tablets per week

Reported reasons for poor adherence are described in Fig. 3. Start-up symptoms, including nausea, vomiting, and dizziness, that lessen after the first month of medication, have been explicitly reported by several trials [6, 8, 13, 31]. Low risk perception is also reported to be a common issue. Many studies report challenges aligning perceived risk with actual risk [13, 14, 32–34]. Participants described concern regarding perceived long term side effects in two studies [32, 33] and poor adherence was partly attributed to dosing regimen in five studies [32, 33, 35–37]. However, a recent study of MSM in Toronto found that high versus low actual HIV risk were more willing to take PrEP (OR 27.11; 95% CI, 1.33 to 554.43) [33].

Societal factors were repeatedly stated as major challenges to maintaining adherence. Governmental and policy factors were mentioned in several contexts, and stigma was reported by many participants in both quantitative and qualitative studies as a barrier to success [17, 38].

### Efficacy

Reported efficacy is highly variable (Table 3), with overall HIV incidence relative risk reduction (RRR) ranging from − 49 to 86% [9, 10, 14]. Both RCTs reporting non-significant RRRs were conducted in the population of young African women [13, 14]. Efficacy among MSM has been consistently high, with recent implementation studies in the UK and Canada both reporting a RRR of 86% in real-life clinical deployment [9, 10]. Heterosexual couples have also achieved high PrEP efficacy with the 2012 Partners study reporting a 75% RRR over 7820 person-years of follow up. Within the single trial in PWID, overall RRR was found to be 48.9 (95% CI, 9.6 to 72.2%).

**Table 3:** Modified Intention to Treat efficacy and effectiveness of studies examining oral PrEP

Characteristics					Outcome
Year	Study name	Population	Regimen	Drug	Efficacy (%, 95 CI)
2010	iPrEx [6]	MSM/TGW	Daily	TDF-FTC	44% (15 to 63)
2012	Partners study [7]	Heterosexual HIV-discordant couples	Daily	TDF-FTC	75% (55 to 87)
				TDF	67% (44 to 81)
2012	TDF2 [8]	Heterosexual	Daily	FTC- TDF	62.2% (21.5 to 83.4)
2012	FEM-PrEP [13]	Heterosexual Females	Daily	TDF-FTC	6% (−52 to 41%)
2013	Bangkok tenofovir study [31]	PWID	Daily	TDF	48.9% (9.6 to 72.2)
2014	iPrEx extension [15]	MSM/TGW	Daily	TDF-FTC	36% (−24 to 67)^a^
2015	VOICE [14]	Heterosexual Females	Daily	TDF-FTC	−4% (−49 to 27)
				TDF	−49% (− 129 to 3)
2015	PROUD [9]	MSM	Daily	TDF-FTC	86% (90% CI 64 to 96)
2015	IPERGAY [10]	MSM/TGW	Event-driven	TDF-FTC	86% (40 to 98)

^a^ Unknown if intention to treat or modified intention to treat. *MSM* men who have sex with men, *TGW* transgender women, *PWID* people who inject drugs, *TDF* tenofovir, *FTC* emtricitabine

**Fig. 2:**
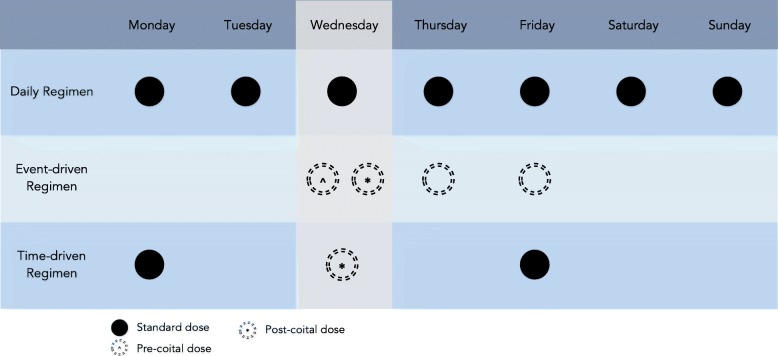
Chart depicting available currently available oral PrEP dosing regimens. The pale column represents a possible HIV exposure event

Three main oral dosing regimen options have been investigated (Fig. 2). Daily dosing is the most frequently tested regimen, with 9 of 11 independent RCTs choosing this route. In event-driven dosing, individuals take two tablets prior to intercourse, followed by single doses 24 and 48 h after the first [10]. Only 1 independent randomised controlled trial, IPERGAY, has evaluated the efficacy of the event-driven regimen so far. Despite only 43% of participants meeting optimum adherence criteria, IPERGAY found an on-treatment RRR of 86%, on par with the daily regimen. Furthermore, mean pill use was halved with participants only using 15 pills per month, versus 30 per month with daily dosing. Time driven dosing, where individuals take pills twice weekly with a post-intercourse boost, has also been evaluated for safety and adherence [35, 36, 38]. This success was replicated in an open label extension, which found 97% (95% CI 81 to 100) effectiveness [22].

When analysis is limited to participants with detectable study-drug serum concentrations, efficacy is higher without exception [6–8, 10, 31] reaching 92% in the iPrEx study subgroup. In an open label extension of iPrEx, no participants with plasma TDF concentrations consistent with 4 or more pills per week underwent seroconversion [15]. The two trial arm participants to undergo HIV seroconversion returned 60 and 58 pills out of 60 for pill count, so were seemingly non-adherent.

### Emergence of resistance in patients

Several trials report individuals who were infected between enrolment and randomisation [7, 9, 14], or had missed diagnoses of pre-existing HIV infection [8], and were later randomised to receive PrEP [Table 4]. Fem-PrEP reported 4 cases of resistance to FTC (3 cases of the M184 V mutation, 1 case of the M184I mutation) in trial-arm participants. VOICE reported FTC resistance in 2 women infected between enrolment and randomisation (2 cases of M184 V/I), and 1 woman infected post-randomisation (M184 V mutation), all in the trial arm. The 2012 TDF2 trial reported the K65R, M184 V, and A62V mutations in 1 of 10 trial-arm participants infected with HIV. That individual had an unrecognised HIV infection at baseline. The PROUD trial reported FTC resistance in 2 individuals assigned to the immediate arm who were infected with HIV at baseline or 4-weeks (66.6%), but no resistance in either of the 2 participants infected later on. Only 2 cases of resistance have been reported in HIV infected individuals assigned to placebo/comparator groups.

**Table 4:** Cases of resistance have been reported in several oral PrEP studies

Study Name	Trial arm			Placebo/comparator arm		
	Total HIV infections	Cases of resistance	%	Total HIV infections	Cases of resistance	%
Fem-PrEP [13]	34	4	11.7	39	1	2.56
TDF2 [8]	10	1	10	26	1	3.85
VOICE (TDF-FTC arm) [14]	61	3	4.92			0
PROUD [9]	5	2	40	0	0	0

## Discussion

### Efficacy and effectiveness

This review discusses adherence to oral PrEP in the context of efficacy data from previous studies. We found that oral TDF and TDF-FTC PrEP for the prevention of HIV in humans is efficacious and effective in a variety of scenarios. Two recent trials within MSM populations in the UK and France/Canada report 86% effectiveness (90% CI, 64 to 96%) [9], and 86% efficacy (95% CI, 40 to 98%) [10] in daily and event-driven regimens respectively. Additionally, PrEP is efficacious in serodiscordant heterosexual couples [7] (efficacy 75%; 95% CI, 55 to 87%).

However, two large trials in heterosexual women failed to demonstrate efficacy [13, 14]. Whilst adherence was low in both studies, as inferred from plasma drug levels, concerns have been previously raised regarding the differential distribution of antiretroviral (ARV) components within rectal and cervical mucosae [20]. Rectal tissue concentrations of TDF are two orders of magnitude greater than in cervical tissue at the same dose, suggesting that equal dosing for men and women may result in insufficient mucosal concentrations to prevent HIV infection in females. Atypical vaginal microbiota have been proposed to decrease the effectiveness of PrEP and increase the risk of HIV acquisition, possibly by increasing ARV metabolism or by weakening the cervicovaginal barrier [39]. However, a post-hoc analysis of the Partners study found that oral PrEP was equally efficacious among woman with bacterial vaginosis as without, and furthermore was not significantly different with the detection of *G. vaginalis* or *Bacteroides* spp. morphotypes [40]. This suggests that oral PrEP formulations do not require testing for bacterial vaginosis or treatment to ensure protection from HIV acquisition.

### Adherence

Adherence to oral PrEP varies greatly between trials and study populations. We found that adherence was consistently high when measured via self-report, pill count and electronic methods, but generally lower when assessed via plasma drug concentrations of TDF and/or FTC. Furthermore, ‘detectable plasma TDF’ rates are frequently reported, however the lower limit of plasma drug detection corresponds to fewer than two pills per week (very poor adherence), making interpretation challenging. While many participants over-report adherence to PrEP, it is unclear whether this is intentional or not. This may be due to social desirability bias as participants in the trials frequently receive adherence counselling, and therefore are well aware of the importance of compliance to PrEP. Comparison of adherence between trials is further complicated by the large variety of adherence measures available, with different methods used within measures themselves. For example, self-report methodology varies from daily SMS reports to monthly interviews, whilst pill count methodology includes unannounced home visits, MEMS, and pharmacy counts amongst other strategies. Therefore, comparability is limited between studies and study populations.

Promisingly, a succession of recent papers and conference abstracts report high levels of real-world adherence [22–24]. One open label intervention of daily PrEP found 80% of participants had protective plasma drug levels at 48 weeks [23], and an open label investigation of event-driven PrEP yielded detectable plasma drug levels in 71% of participants at 6 months [22]. However, Computer Assisted Structured Interviews determined that on-demand PrEP was only used at the correct dose in 50% of sexual intercourses. Whilst interviews may suffer from self-report and recall bias, one might expect adherence to be overestimated, rather than underestimated, due to social desirability bias. Although there is currently insufficient data to justly compare on-demand and daily regimens, this disparity should be noted for further investigation.

### Reasons for non-adherence

**Fig. 3:**
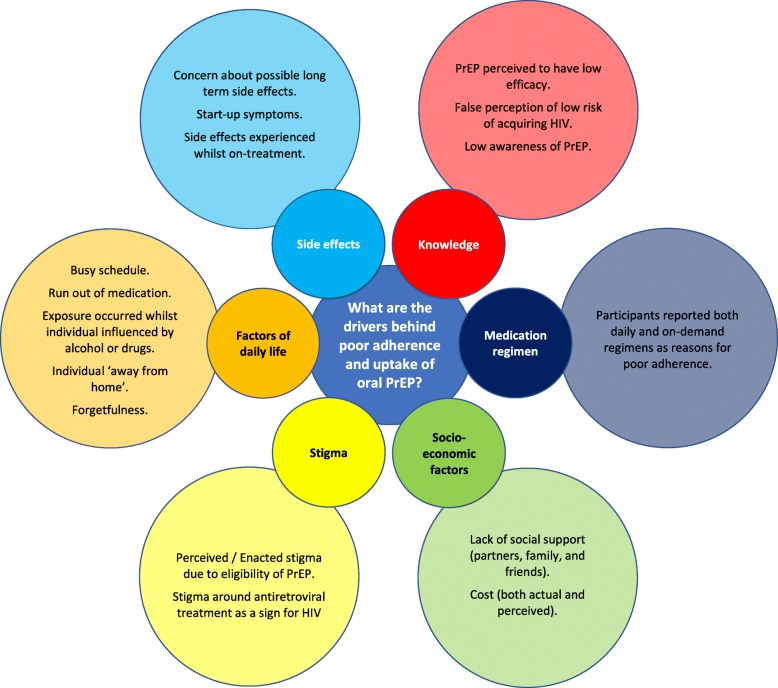
Grouped reasons reported for poor adherence to oral PrEP that were found in studies included in this article, and for high risk individuals declining medication

The reasons reported for non-adherence (Fig. 3) are broad, reflecting the wide variety of populations and settings in which trials have been performed. Common qualitative reasons for poor adherence included participant low risk perception, side-effects, perceived stigma and dosing regimen incompatibility. These findings are consistent with reports from individual trials, which note that start-up side effects are frequent [6, 8] and may have influenced adherence. However, the Bangkok Tenofovir Study reported that nausea and vomiting were start-up symptoms which abated after the first couple of months [31]. However, of trials reporting dosing regimen as a reason for low adherence, three used a daily regimen and two used an on-demand regimen, implying limited acceptability regardless of daily or on-demand dosing regimen. However, comparatively little research has been performed using on-demand regimens, therefore further research is required to explore the differences in acceptability between daily and on-demand regimen. Recent modelling suggests weekly oral dosing with controlled release formulations may lead to improved adherence [41], implying that long-acting PrEP formulations may provide some solutions to poor acceptability of current dosing regimens.

### Stigma

“Stigma remains the single most important barrier to public action [against HIV]”, wrote ex-UN Secretary General Ban Kai-Moon in 2008 [42]. This statement is unfortunately still just as relevant in both low- and high-income settings, and has important implications for PrEP initiatives worldwide [43]. Qualitative investigation of PrEP trials has elicited both social and self-stigmatisation as instrumental challenges for participant adherence. Interviews with participants from the failed VOICE trial found that it was important for women from South Africa, Zimbabwe and Uganda to be perceived as healthy by the community [44]. Taking medication associated with being HIV positive did not align with their narrative of health through self-stigmatisation, which may have detrimentally affected adherence. Furthermore, participants were understandably concerned that community misunderstanding regarding PrEP could cause friends and family to believe that they were HIV positive [45]. Some participants resorted to hiding the medication and pill bottles, however the conspicuous physical characteristics of the tablet were hard to explain. In the most severe cases, participants experienced extreme reactions from their close family, even resulting in spouse or partner separation [44, 45].

### Risk perception and knowledge

Low risk perception is a common issue within PrEP trials. Despite adherence counselling, a large proportion of women (> 70%) from Kenya, Tanzania and South Africa reported themselves as at low or no risk of HIV in the failed FEM-PrEP trial [13]. Low risk perception was also a common reason for MSM declining PrEP in the United States PrEPARE trial [32], despite all eligible individuals belonging to a population at actual high risk. Reasons for low risk perception are unclear, although may relate to generally poor HIV education, which is often neglected in sex education [46–48]. PrEP will need to be delivered within a comprehensive package, including regular HIV awareness and PrEP adherence counselling if sufficient adherence for success is to be maintained.

### Decision making power

PrEP is often prescribed to individuals who live in difficult circumstances. MSM, transgender, sex worker and PWID populations carry burdens of HIV disproportional to their size, and are at risk of being left behind in HIV prevention [49]. Stigma and criminalisation further marginalises these groups in many countries [3, 4]. The prospect of social ostracism and prosecution introduces further structural barriers to accessing healthcare services, reducing PrEP uptake and adherence. In a study of young MSM of colour in the USA, 39% had been kicked out of their home due to their sexual orientation and 43% had spent at least one night on the street [50]. These factors, combined with the prevalence of transactional sex, mean that young women and MSM, particularly transgender women, are often subject to abuse [5, 50] and frequently lack decision-making power over their bodies when it comes to sexual encounters [51]. These structural and social barriers, which reduce agency, can generate considerable difficulty in maintaining sufficient adherence to the dosing regimen and in accessing health services [3]. Despite this data previous trials within MSM populations have been surprisingly successful compared with young heterosexual women. This review cannot resolve this difference, but considering gender perspectives within differing populations may offer some insight, particularly by considering the relation of cultural gender roles to decision-making power.

### Drug resistance

With poor adherence a frequent issue in PrEP users, the question of drug resistance is of elevated concern. A full review of drug resistance in PrEP lies beyond this review, but most published RCTs report drug resistance as a rare outcome. Due to the rarity of resistance, it is also currently difficult to quantify the risk. However, it does appear to be more frequent among individuals receiving PrEP.

The infection of individuals between enrolment and randomisation [7, 9, 14], or missed diagnoses of pre-existing HIV infection [8], meant that these participants were likely exposed to high drug concentrations whilst in the acute phase of HIV infection. This was, however, very rare in the RCTs. As HIV serology assays that are often used cannot detect HIV infection during the acute phase, this remains a challenge for PrEP programmes in low income settings. HIV-RNA detection can be performed at enrolment to ensure that PrEP is not prescribed to any individual who recently acquired HIV, which may be cost effective and even cost-saving in higher prevalence populations [52]. Regardless, it is difficult to know whether mutations are due to prescribed medication, or due to previous exposure without healthcare worker consultation. We also note an MSM individual who was fully adherent to TDF-FTC PrEP was recently reported to have been infected with a resistant strain of HIV [53]. This provided the first compelling evidence of breakthrough infection despite good adherence to oral PrEP by drug-resistant HIV-1.

From this data, it seems that PrEP is not associated with a large risk of drug-resistance developing. The low drug plasma concentrations associated with poor adherence appear to confer a low risk of resistance should HIV infection occur, whilst high plasma concentrations in adherent individuals make resistance development unlikely through successful inhibition of viral replication. However, with wide scale PrEP use around the corner, resistance may soon become a greater issue, especially in developing countries where follow-up and routine monitoring is more difficult. Furthermore, infection by resistant strains remains a rare possibility and individuals who are infected with HIV whilst truly adherent to PrEP may propagate resistant strains. Thorough disease history and clinical examination could help to detect acute phase HIV. When acute phase HIV is suspected, PrEP can be delayed to ensure a reliable negative HIV serology before initiation or HIV-RNA analysis can be performed in settings where this is available. Reasonable care should be taken to ensure participants are not infected with HIV prior to PrEP initiation.

### Challenges in clinical practice

Current knowledge relating to oral PrEP suffers from knowledge gaps. There are few long-term studies relating to effectiveness and adherence, and while some trials report that adherence is stable over time [15], others suggest a long-term decline [17, 18, 54]. This is particularly important for oral PrEP due the importance of good adherence for its protective effect. It is often challenging to trace and maintain interaction with populations most at risk of HIV acquisition. This is critical for the success of PrEP due to the necessity of regular pill distribution and HIV/STI testing. To further complicate matters, a recent study in Bangkok found little association between participants *intending* to take PrEP and *actual* adherence at 1 month [55]. It is important to demonstrate that PrEP adherence can be maintained in key populations over time for it to be effective.

Effective methods of encouraging adherence are likely to be as varied as the populations themselves. Success was reported in a US community based programme [50] through four key strategies. First, PrEP was delivered as a key component within a comprehensive prevention package, from a place often visited by the population (e.g. young MSM). Secondly, high contact frequency (weekly) was maintained. Thirdly, the package promoted all aspects of a healthy lifestyle., and finally aimed to further empower individuals through optional weekly workshops focussing on life-skills. Linked with a comprehensive strategy, peer navigators, who aim to solve individual barriers to PrEP, are being evaluated as an option to maintain adherence and retention [56]. One recent study (SPARK) also found high adherence rates at 3 months in conjunction with a comprehensive sexual health intervention, supporting the feasibility of incorporating PrEP adherence counselling into existing frameworks [57].

This promising model could feasibly be adapted for use in other populations. Multi-modal intervention models are effective in maintaining medication adherence for other conditions, but it is recommended that programmes are designed to allow evaluation of individual components [58]. There is already evidence that text messaging is highly acceptable and may improve retention in PrEP programs [59]. Smartphone penetration is also high in many countries, such as the UK where 91% of 18 to 34 year olds own a device [60], and Sub-Saharan Africa where penetrance is expected to exceed 50% by 2020 [61]. This could present an opportunity for innovative adherence solutions. Apps could be designed to display medication reminders, allow adherence self-reporting, and even to incentivise good adherence through reward.

The increasing online availability of generic PrEP, which is accessed and used by individuals without a doctor’s prescription and without proper prior HIV screening, presents a real challenge. If the user cost of accessing PrEP on prescription exceeds the cost of purchasing generic versions online, then individuals are likely to take PrEP acquisition into their own hands. In France, the first and only European country to offer PrEP through public health services, over 60% of on-PrEP MSM access medication via their physician as opposed to less than 30% in other countries [62]. Correspondingly, less than 10% of on-PrEP French MSM access medication online as opposed to over 40% in other European countries [62]. Unmonitored PrEP usage could result in risk to users from adverse effects due to excessive dosing, and HIV infection due to insufficient dosing, whilst simultaneously accelerating the development of resistance if new infections are undiagnosed. It may also present a public health risk if high-risk individuals using unmonitored PrEP perceive themselves at lower risk of HIV, and subsequently do not attend HIV testing services as frequently. However, 31 European countries still identify cost of medication and service delivery as a major barrier to PrEP implementation, despite this unique opportunity to target HIV transmission in the most high-risk groups [62]. Despite this, recent modelling research in the UK suggests cost-effectiveness and long term cost-saving benefits across a wide range of PrEP introduction scenarios for the MSM population [63]. Cost savings depended on both the eligible population and the risk of HIV acquisition, so long term cost-effectiveness is likely to be even greater in locations with higher HIV prevalence. Notably, cost-effectiveness was highly time sensitive, suggesting that policy makers must consider PrEP over lifetimes, and not merely the political cycle.

### Limitations

Despite best efforts to ensure a comprehensive search, there may be eligible studies that we failed to include. We made efforts to contact authors of soon to be released trials, but not all authors were contactable. Secondly, the conclusions we draw are only as good as the data provided. There is reason for concern over the use of pill counts and current electronic monitoring methods as measures of PrEP adherence due to the varying concordance with blood plasma drug concentrations. Furthermore, this review is limited to discussion of oral PrEP adherence in the context of efficacy. Long-term safety, cost effectiveness, HIV drug resistance and sexual behaviour trends are not evaluated or discussed in detail. Finally, whilst this paper discussed adherence in detail, it must be noted that adherence is just one step in the broader PrEP retention continuum [16].

## Conclusions

Oral PrEP can be effective for the prevention of HIV. Some interventions have achieved high adherence and clinical effectiveness among MSM. However, further exploration of the biological and sociocultural reasons for poor adherence in other populations such as women is required. Cheap and accurate methods of long term adherence monitoring, such as urine testing, require development and validation. Interventions must be designed with user-appropriateness in mind, considering the sometimes unpredictable lives of at-risk individuals at the fringe of society in addition to those in the centre. Flexible medication delivery models and extended release PrEP formulations will likely play an important role in catering to these needs, and further research will be needed to design and prove these methods. Moreover, efforts should be taken to challenge the stigma, marginalisation and prosecution of minority groups, such as sex workers, PWID, and MSM, both within the community and at governmental level. Drug resistance to PrEP is still rare, but sufficient data to fully quantify the risk of resistance is likely to only be available once widespread use in lower income settings and at larger scale is achieved. Finally, the cost-issues for using preventive ARVs must be dealt with at national level in many countries.

## Abbreviations

ART: Antiretroviral Therapy
ARV: Antiretroviral
CDC: Centres for Disease prevention and Control
FTC: Emtricitabine
MEMS: Medication Event Monitoring System
MSM: Men who have Sex with Men
PrEP: Pre-Exposure Prophylaxis
PWID: People Who Inject Drugs
RCT: Randomised Controlled Trial
RDBPCT: Randomised Double Blind Placebo Controlled Trial
TDF: Tenofovir disoproxil fumarate
TGW: Transgender Women
UN: United Nations
WHO: World Health Organisation
